# Supplementary material for: Scalable and accurate deep learning with electronic health records
Source: NPJ Digit Med. 2018 May 8;1:18. doi: 10.1038/s41746-018-0029-1 (PMC6550175; doi:10.1038/s41746-018-0029-1)
Supplement: Supplementary file 1 — Supplementary Materials [file 41746_2018_29_MOESM1_ESM.pdf]

Supplementary Materials

Scalable and accurate deep learning for electronic health records

Alvin Rajkomar*[1,2], Eyal Oren*[1], Kai Chen[1], Andrew M. Dai[1], Nissan Hajaj[1], Michaela Hardt[1], Peter J. Liu[1], Xiaobing Liu[1], Jake Marcus[1], Mimi Sun[1], Patrik Sundberg[1], Hector Yee[1], Kun Zhang [1], Yi Zhang[1], Gerardo Flores[1], Gavin E. Duggan[1], Jamie Irvine[1], Quoc Le[1], Kurt Litsch[1], Alexander Mossin[1], Justin Tansuwan[1], De Wang[1], James Wexler[1], Jimbo Wilson[1], Dana Ludwig[2], Samuel L. Volchenboum[4], Katherine Chou[1], Michael Pearson[1], Srinivasan Madabushi[1], Nigam H. Shah[3], Atul J. Butte[2], Michael Howell[1], Claire Cui[1], Greg Corrado[1], and Jeff Dean[1]

[1]Google LLC, Mountain View, California
[2]University of California, San Francisco, San Francisco, California
[3]Stanford University, Stanford, California
[4]University of Chicago Medicine, Chicago, Illinois

March 15, 2018

Contents

1 Data Representation **1**
1.1 Embeddings 2

2 Description of Inclusion Criteria and Outcomes **3**

3 Model Variants **5**

4 Methods for All Techniques **9**

5 Baseline Models **10**

1 Data Representation

Data from each electronic health record was imported into a new schema based on the open-source Fast Healthcare Interoperability Resources (FHIR) resource standards. We populated data into elements from the following resources because they were available in both datasets: Patient, Encounter, Medication, Observation (e.g. vital signs and nursing documentation), Composition (e.g. notes), Conditions (i.e. diagnoses), MedicationAdministration, MedicationOrder, ProcedureRequest, and

Procedure. We imported the data directly from the health system, meaning we did not harmonize elements to a standard terminology or ontology. If a health system included multiple terminologies, like a site-specific coding scheme and an RxNorm code (a common medication coding scheme), we imported both. The only exceptions were for diagnoses/procedures, which we mapped to ICD9/10 and CCS categories if the health system did not already include them (e.g. for CPT codes), and for elements that were used to define the primary outcomes, as described in the main manuscript.

In the electronic health record datasets, there was a category of data referred to as "flowsheets," which correspond to many structured data elements in clinical care, like vital signs and nursing documentation. Depending on workflows, data may be collected at the bedside, like a temperature reading and then entered in the EHR later. This documentation provides (at least) two timestamps – when the data was technically collected (recorded time) and when it was entered (entry time). We specifically used the entry time in the EHR because especially during emergent situations, the recorded times are estimated. We found that using the recorded-times significantly improved prediction accuracy, but refrained from using them as the data is not actually available in the EHR at that point-in-time.

FHIR is increasingly used to exchange healhcare data, and when available can be used as model input directly as described in the manuscript. In this study however, data was originally collected from vendor-specific database tables. The mapping from those tables to FHIR was manual, but straightforward, because most tables and fields map directly to existing resource types and attributes in FHIR, and because no data harmonization is required in our approach. To incorporate EHR data that did not map straightforwardly to an existing FHIR resource type or attribute, FHIR extensions can be defined.

1.1 Embeddings

In this section we explain the process of mapping FHIR resources to tokens, how individual tokens were embedded, and how time was entered into the data-representation.

Each FHIR resource is composed of multiple elements, which we refer to as features. For example, a FHIR resource corresponding to a medication order would have an element (feature) corresponding to the name of the medication ordered and another element corresponding to the RxNorm code of the medication. The value of each feature (e.g. "Aspirin") was assigned either a single unique token (for singular elements) or sequence of tokens (for free-text elements). Notes and other text features (text descriptions of codes or names of medications, laboratory tests, and procedures) were first tokenized (split on whitespace and punctuations etc.) and then mapped to a sequence of tokens.

Numeric values were represented differently in the model architectures. In general, two ways were used: the first was to concatenate the name, value, and unit (e.g. "Hemoglobin 12 g/dL") and assign this to a unique token. A second approach was to concatenate the quantile of the value (calculated specifically to the type of data-element: for example, 12 might be the median for hemoglobin but 99-th percentile for hemoglobin-a1c) to the unit and assign this to a unique token.

The unique tokens of a feature (e.g. medication codes or words in a note or observation percentiles) were then individually mapped to a d-dimensional floating-point embedding vector $\vec{e}$ with d either tuned as a hyperparameter or derived from the number of unique tokens. As long as a token occurred at least twice in the training data-set it would receive its own embedding vector (for observation code and value pairs, the token had to appear at least 100 times). Less frequent tokens were hashed and mapped to a small number of out-of-vocabulary embedding vectors. All embeddings are randomly initialized.

Supplemental Figure 1 illustrates part of the preceding discussion.

How the time associated with each resource was mapped to tokens differed for the model architectures, however they all rely on the difference to the time of prediction in seconds, referred to as delta-time. The recurrent neural network value took the logarithm of this delta-time rounded to the next integer and capped at a maximum. The feed-forward network bucketized the delta-time into exponentially increasing buckets of $1, 2, 4, 8, \ldots$ days. The age in years of the patient was also similarly bucketized, embedded and concatenated to the output of the final layer. The boosting model learned thresholds of the delta-time as a way to discretize it.

2 Description of Inclusion Criteria and Outcomes

Inclusion Criteria

Inpatient encounters were defined as followed: 1. Encounter was confirmed as complete or non-cancelled 2. Encounter had a start and end time 3. Encounter class was defined as inpatient as defined in dataset 4. Administrative encounters were excluded (e.g. no primary diagnosis was documented); these encounters accounted for less than 1 percent of hospitalizations in the data received.

The following services were included in the Medical-Surgical Cohort: General Medicine, Cardiology, Neurology, Critical Care Medicine, Hematology, Oncology, Hepatobiliary Medicine, Medical Specialties, General Surgery, Colorectal Surgery, Otolaryngology, Gynecology, Gynecology-Oncology, Neurosurgery, Oral-Maxillofacial surgery, Orthopedics, Plastic Surgery, Thoracic Surgery, Transplant Surgery, Urology, and Vascular Surgery.

Cohort Definitions

We made the following modifications of the CMS cohort definitions[1] to ensure that every primary diagnosis was listed in a cohort.

We added CCS code 150 (alcoholic liver disease) to the Medicine Cohort.

We created the following new Cohorts: Obstetric Cohort containing CCS codes 176-196. Rehabilitation Cohort containing CCS code 254 Injury and Poisoning Cohort containing CCS codes 260 and 2601-2621.

Determining Unplanned Readmission

We implemented the logic used by CMS to define planned readmissions[1]. The logic evaluates whether admissions were for reasons that are defined to be planned (e.g. bone marrow transplants and chemotherapy), and it distinguishes between surgical procedures that were accompanied by an acute condition (e.g. acute cholecystitis) or non-acute condition, which were defined to be unplanned or planned, respectively.

In the 2016 version of the CMS rules, some criteria were defined by a mix of CCS and ICD-9 procedure and diagnosis codes. Given that some hospitalizations only had ICD-10 diagnoses and procedure codes, we mapped the ICD-9 CMS codes to ICD-10 and then applied the rules. We used the mapping tables provided by the National Bureau of Economic Research[2].

Fewer than 1 percent of hospitalizations did not have a primary diagnosis marked in the raw data. Based on a review of a random sample of these hospitalizations, these encounters lacked clinical data about events in the hospitalization and were therefore not included but they did have administrative

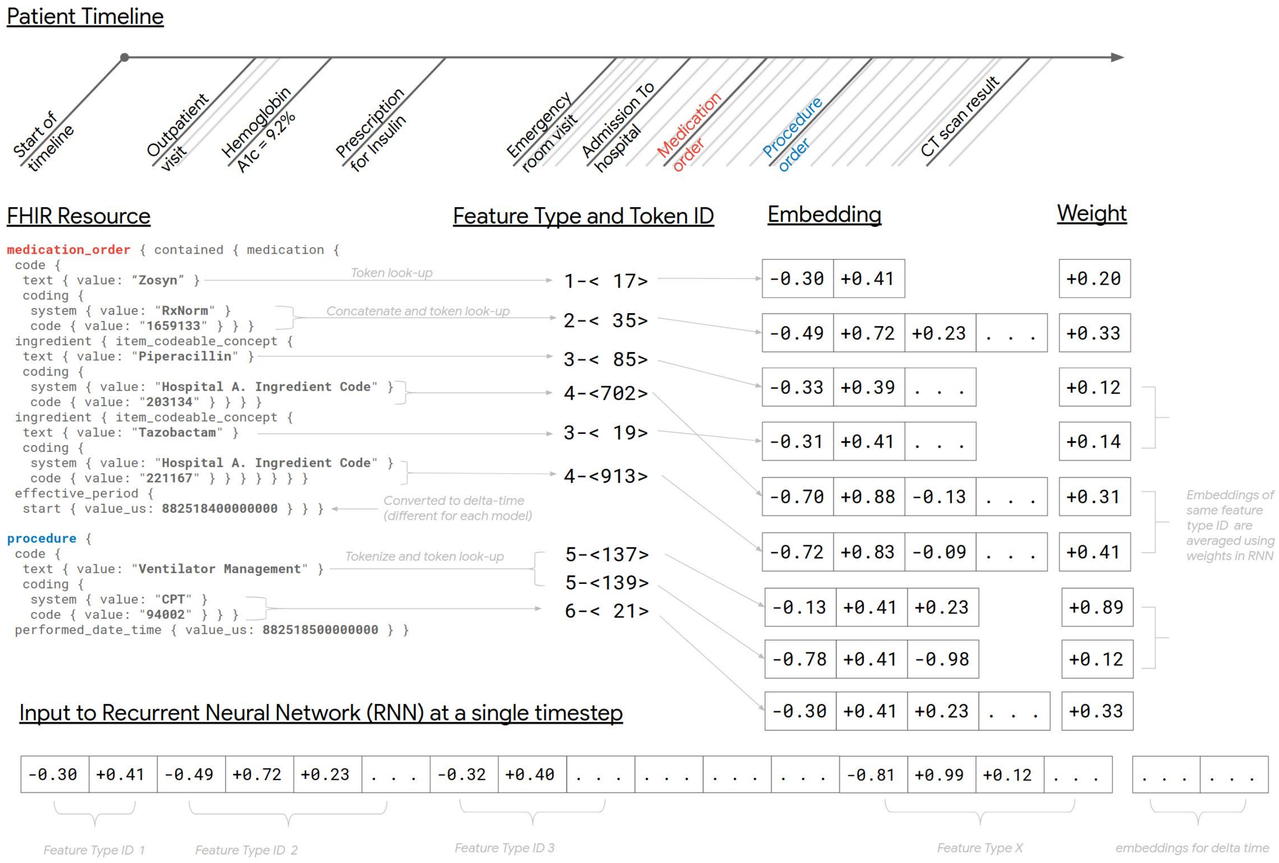

Figure 1: Conversion of FHIR resources to embeddings. The patient timeline contains a temporally ordered sequence of FHIR resources, visually depicted as flags, and details for the resources corresponding to a medication order (in red) and procedure order (in blue) are depicted below the timeline. Each resource is composed of features, like a medication_order.code.text or procedure.coding. Features are mapped to token IDs that are specific to a feature type (depicted in the figure as having a feature type ID). For coding systems, the coding system is concatenated with the code (e.g. "Hospital A. Ingredient Code 203134") before being given a token ID. Note that in the feature of procedure.code.text, the phrase "Ventilator Management" is separated into two separate tokens, one for each word. Each token ID is associated with an embedding and weight (in the recurrent neural network [RNN]). The embeddings and weights are randomly initialized and updated through the training procedure. For the RNN, if two tokens exist for the same feature in a resource (e.g. 5-137 and 5-139), the corresponding embeddings are averaged with weights, as described in the Supplement text. Note that the time-stamp is used in relation to the prediction time to calculate the delta-time, which is subsequently dealt with differently by each model architecture. For the RNN, the embeddings for all features at a given time-stamp are concatenated.

data that indicated that these were unplanned admissions. After confirming with the respective partner sites, we treated these cases as ineligible to be index discharges given missing data but were considered unplanned admissions. They were excluded from the mortality and diagnosis prediction tasks.

Discharge Diagnoses

The discharge diagnoses include the primary and secondary diagnoses for the entire hospitalization, including conditions diagnosed (and documented) about during the hospital stay. However, the codes are often physically entered at the end or even after the end of a hospitalization (sometimes by a professional biller), so we timed all the diagnoses of occurring the moment just after discharge. To ensure no leak of information, we timed the occurrence of billing codes of a hospitalization to occur immediately after a discharge; therefore, the prediction of diagnoses at discharge time does not have access to these codes.

3 Model Variants

Weighted Recurrent neural network model (RNN)

In the RNN model, sparse features of each category (such as medication or procedures) were embedded into the same d-dimensional embedding. d for each category was chosen based on the number of possible features for that category according to the heuristic $6\alpha_d \sqrt[4]{V}$ where α_d is a hyperparameter and V is the vocabulary size. The embeddings from different categories are concatenated together. We then combine the embeddings in a novel way: for the same category and same time, the embeddings are averaged according to an automatically learned weighting.

The sequence of embeddings were further reduced down to a shorter sequence. Typically, the shorter sequences were split into time-steps of 12 hours where the embeddings for all features within a category in the same day were combined using weighted averaging. The weighted averaging is done by associating each feature with a non-negative weight that is trained jointly with the model. These weights are also used for prediction attribution. The log of the average time-delta divided by a factor (controlled by a hyperparameter) at each time-step is also embedded into a small floating-point vector (which is also randomly initialized) and concatenated to the input embedding at each time-step.

This reduced sequence of embeddings were then fed to an n-layer Recurrent Neural Network (RNN), specifically a Long Short-Term Memory network (LSTM)[3]. An RNN consists of a sequence of directed nodes. Embeddings are fed to the RNN one at a time and for each time-step, each node computes its activation as a nonlinear function of the input embedding. Each subsequent node receives as input the previous node's activation and the embedding for that time-step. The LSTM extends the RNN by adding 3 gates, an input gate, output gate, forget gate to determine what information to pass on to the next node relative to the previous node's activation and the current time-step's embedding. Each node in the LSTM computes an hidden state vector and cell state vector.

The LSTM is defined by the following set of equations where W and U corresponds to weight matrices, b to biases and the subscript and variable f, i, o represent the forget, input and output gates. h_t represents the hidden output at time t, x_t represents the input at time t and c_t represents

the cell state at time t. σ_g represents the sigmoid function and σ_c the hyperbolic tangent.

$$
\begin{aligned}
f_t &= \sigma_g(W_f x_t + U_f h_{t-1} + b_f) \\
i_t &= \sigma_g(W_i x_t + U_i h_{t-1} + b_i) \\
o_t &= \sigma_g(W_o x_t + U_o h_{t-1} + b_o) \\
c_t &= f_t \cdot c_{t-1} + i_t \cdot \sigma_c(W_c x_t + U_c h_{t-1} + b_c) \\
h_t &= o_t \cdot \sigma_c(c_t)
\end{aligned} \tag{1}
$$

The hidden state of the final time-step of the LSTM was fed into an output layer, and the model was optimized to minimize the log-loss (either a logistic regression or softmax loss depending on the task). We applied a number of regularization techniques to the model, namely embedding dropout, zoneout[4], LSTM hidden dropout and variational RNN dropout[5]. We also used a small level of L2 weight decay, which adds a penalty for large weights into the loss. We trained with a batch size of 128. Finally, we optimized everything jointly with Adagrad[6] for the binary tasks and Adam[7] for the multilabel tasks. For the multilabel tasks we also multiply the input to the LSTM at the final-timestep with a special EOS embedding. We trained using the TensorFlow framework on the Tesla P100 GPU. The hyperparameters were found via a Gaussian-process based hyperparameter search on each dataset's validation performance.

The hyperparameters used for the model for Hospital B are listed below. p_k denotes keep probability:

Hyperparameter	Mortality/Readmission	Discharge diagnoses
Embedding size multiplier α_d	0.5736	1.0
Dense embedding size	100	200
Gradient clipping	11.16	0.1245
Embedding dropout p_k	0.4410	0.7542
Hidden dropout p_k	0.4740	0.8641
L_2 penalty	0.000001566	0.0
Learning rate	0.4375	0.003688
LSTM hidden size	279	518
Variational input p_k	0.9768	0.9290
Variational output p_k	0.9747	0.8780
Variational recurrent p_k	0.9976	0.9958
Variational vocabulary p_k	0.5017	0.7266
Zoneout p_k	0.9489	0.5628
Deltatime embedding size	20	30
Deltatime factor	0.41	1.0

Feedforward Model with Time-Aware Attention

To the sequence of embedding, $E_i, i = 1, \ldots, n$ we added an additional prior embedding to the sequence E_0 with the associated $\Delta_0 = 0$. For every embedding $E_i, i = 0, \ldots, n$ we created an attribution logit α_i using the process described below. Those logits were converted to weights β_i using softmax,

$$\beta_i = \frac{e^{\alpha_i}}{\sum_{j=0}^{n} e^{\alpha_j}} \tag{2}$$

We then took the d dimensional vector of the weighted sum, $E = \sum_j \beta_j E_j$, along with the scalars $\log(n+1)$ and $\log(\sum_j \beta_j^2)$, and entered them into a feedforward neural network whose attributes (e.g. number and dimensions of the layers) were determined by hyperparameter tuning.

For the attribution logits, we used a bank of k functions, $A_1(\Delta), \ldots, A_k(\Delta)$, where each A_j had one of the following forms (typically not all forms in the same model):

- $A(\Delta) = 1$ (constant);
- $A(\Delta) = \Delta$;
- $A(\Delta) = \log(\Delta + 1\text{day})$;
- $A(\Delta) =$ Piece-wise linear function with predetermined inflection points (based on exponential backoff) and learned slopes.

We defined a k dimensional projection of the embedding by learning a $k \times d$ dimensional matrix P, and for every $i = 0, 1, \ldots, n$ multiplying it with E_i to get the k scalars $p_{1,i}, \ldots, p_{k,i}$. We then defined the attribution logits to be

$$\alpha_i = \sum_{j=1}^{k} p_{j,i} A_j(\Delta_i) \tag{3}$$

The embedding dimension, d, ranged from 16 to 512. The number of layers of the feedforward network ranged from 0-3 with the width of the networks from 10 to 512.

Boosted, embedded time-series model

For the boosting model, bigrams, trigrams and 4-gram tokens were created from all tokenized features. For each feature tuple of the token name, value, and time-delta, we algorithmically created (described below) a set of binary decision rules that partitioned examples into two classes.

There were ten types of decision rules.

- The first was whether a variable, X, existed at any-point in a patient's timeline.
- The second was whether a variable X existed more than C times in a patient's timeline. C was randomly picked from the range of integer values possible for each variable in the dataset.
- The third introduced the time sequence nature of the variable: was variable X greater or lower than threshold V at any time $t <$ T (i.e. $x > V$ and $t < T$; or $x \leq V$ and $t < T$). Again, V and T were picked from the space of possible values in the dataset.

  The fourth was a modification of the third rule, but rather than a simple binary cutoff, it was a weighted sum of of the number of times that rule ($x < V$) was satisfied, with the weights determined by a Hawkes process response with a time decay factor of T. A binary rule was created by examining if this weighted sum was greater than the activation A, that is $A_{instance} > A_{template}$, where $A_{template}$ is selected from a random user. Again, we use random

selection of a particular template instance to select V and T. Then A is computed from the instance by

$$A = \sum_i I\{x_i > V\} e^{\frac{-t_i}{T}} \tag{4}$$

- The fifth, six, and seventh rules were created by determining if the minimum, maximum, and average of variable X was greater than V in time $t < T$.
- The eighth and ninth type of rule captured changes in lab values over time (e.g. the decrease in blood pressure over time). In particular, the eighth predicate checked the velocity, that is if there is a change in a variable divided by a time window that is greater or lower than a threshold V. The ninth predicate checked if the difference in values within time T is greater or lower than a threshold V.
- The tenth type of rule consists of conjunctions of previous predicates (e.g. does X_1 exist and does the count of X_2 exceed C_2),We call these decision list predicates as, to preserve interpretability, they only encompass the true branches of a decision tree. The conjunctions are mined by picking the best predicate in a random selection of predicates, then, conditioned on the best predicate, the a second one that also maximizes the weighted information gain with respect to the label.

The actual instances of each rules, including the selection of variables, value thresholds and time-thresholds were generated by first picking a random patient, random variable X, and a random time T in the patient's timeline. V is the corresponding value of X at time T and C is the counts of times X occurs in the patient's timeline.

Every binary rule, which we refer to as a binary predicate, was assigned a scalar weight, and the weighted sum was passed through a softmax layer to create a prediction. To train, we first created a bias binary predicate which was true for all examples and its weight was assigned as the log-odds ratio of the positive label class across the dataset.

Next, we used rounds of boosting to select predicates. In each round, we picked 25,000 random predicates from random patients in a batch of 500 patients. Importance-weighted information gain with respect to the label was calculated for each and the top 50 predicates were picked. Additionally, for each of those top 50 predicates, 50 more secondary predicates were selected using the same information gain criteria, conditional on the primary predicate holding true. The best predicate and second corresponding predicates were then joined together to create 50 more conjunction predicates for a total of 100 predicates per round. Weights of these predicates were fitted using logistic regression with L_1 regularization. We then applied the model to all examples in the training dataset to create prediction probabilities Q. Each example was then given an importance weight of $|Label - Q|$.

In the next round, we selected 25,000 new random predicates by sampling examples according to the importance weight. The top 50 by information gain (and 50 more secondary ones) were added to a new logistic model which included the predicates from the previously determined predicates. The weights of all predicates were re-calculated (i.e. not just the new predicates), which is known as totally corrective boosting.

We used 100 rounds, so in total 100,000 predicates were selected from a pool of 5,000,000 which were in turn randomly selected from a pool of all possible predicates, which was equal to the product of the number of patients, features, discrete values and time steps. The L_1 regularization was then used, which could further cull away the 100,000 selected predicates to a smaller set.

The final binary predicates were then embedded into a 1024 dimensional vector space and then fed to a feed-forward network of depth 2 and 1024 hidden units per layer with ELU non-linearity. For regularization, Gaussian noise of mean 0 and standard deviation 0.8 was added to the input of the feed forward network. We also used multiplicative Bernoulli noise of p=0.01 (also known as dropout) at the input and output (just before the applying the sigmoid function to the logits) of the feed forward layer. At test time, no Gaussian or Bernoulli noise was used. We optimized everything with Adam. The union of predicates optimized for different tasks (e.g. readmission or different diagnosis codes), were all used together in the final model. These final binary predicates have been mined from different tasks (e.g. for the readmission task, many diagnosis code models might contribute auxiliary binary predicates that they have mined as features for the feedforward network).

4 Methods for All Techniques

Attribution Mechanisms

To explain predictions we implement attribution mechanisms. Inspired by recent results in natural language processing[8], the feed-forward models implement an attention mechanism identifying the locations in a sequence of variables which may have played a significant role in affecting the prediction. Notably, the same variable could be harnessed differently given when it occurred in relationship to other events for a given patient timeline. The RNN models implement a form of weighting that also learns which variables are important for prediction relative to other variables. We use both of these methods to perform attribution.

Illustrating the data that the models attended to is difficult because of the complexity of the data, including thousands of time-steps with tens- to hundreds-of-thousands of tokens, representing a large percentage of all the data that is viewable in a patient's actual EHR record. Moreover, given the correlation of the data (the heart-rate at time x is related to the rate at $x + 1$) and redundancy (the medication order of "norepinpherine" is redundant with the nurse's documentation of the rate to which it is titrated), the models could choose to attend to equivalent data arbitrarily. For visualization purposes only, we re-trained feed-forward models on a single task, mortality, with models using only a single data-type (e.g. notes, medications, observation data) to preclude the models using redundant data among different feature types. These models differ in predictive performance than that of the full models reported in Table 2.

In Figure 4 of the main manuscript, we render the timeline, populating a circle for every time-step where at least a single token exists for that patient. We have shown snippets of select time-steps and highlight the tokens in which the model using that data-type chose to attend it. For tokens with significant attribution scores, we have "smeared" attribution to directly neighboring tokens for visualization purposes. The model outputs attribution for all tokens and we selected the top tokens, which for this particular example, corresponded to a threshold of 0.001, although further research is needed to identify the optimal threshold. For patient privacy reasons, we have obscured information about the dates and times of all tokens, although the relative time has been retained.

Automated Hyperparameter Tuning

There are many design choices to training neural networks that are beyond the scope of this manuscript but are well described elsewhere[9]. The hyper-parameters, which are settings that affect

the performance of all above neural networks were tuned automatically using Google Vizier[10] over thousands of experiments.

Ensembling

For a given prediction task, we could use a variety of algorithms to make a prediction. For example, we could use a sequence model, feed-forward model, and a boosting model, and their predictions would be different on the same example. Ensembling combines the multiple predictions to make a final prediction; this is similar to tallying votes for an election result. We combined the predictions (probabilities) from the three models of the ensemble by averaging.

5 Baseline Models

The first set were constructed using traditional modeling techniques. We used recent literature reviews to select commonly used variables for each task[11–13]. These hand-engineered features are used only in the baseline models; the deep learning models do not use feature selection.

The second set of baseline models were created by using the full set of predictor variables in a simple linear model, to assess the effect of simply including all the variables, which is a common baseline in many machine learning papers. In this model we flattened the entire sequence, ignoring temporal order, and only considered the existence of each predictor variable. For text features such as clinical notes, we considered both the unigram and bigram of the text tokens. For observations such as lab test results, we created two representations. First, for each feature we concatenated the code, value and unit (when available) into a string (e.g. "code:12345 12.6 g/dl"). Second, we concatenated the code, percentile-of-value and unit (e.g. "code:12345 0.75 g/dL," if the raw value 12.6 was the 75-percentile for that observation code in the training set). The percentiles are rounded into 20 buckets. Note that we did not harmonize the medical meaning of the observations, other than giving the model their raw values and percentile buckets. For diagnosis, medication and procedures, we considered all the available codes, such as medication RxNorm and ingredient. The patients' demographic information such as age and gender were also considered. The baseline model is a logistic regression model trained using the Adam optimizer with early-stopping as regularization, since it gave us the best result. We tuned the model on the validation set. We refer to these models as "full feature simple baselines."

The third set of baseline models were very similar to the second set, except that we bucketized the features into five time-buckets, i.e. less than 1 day, 1 week, 1 month or 1 year, or greater than 1 year. This was a simple way to let the model be aware of the time component (e.g. whether a treatment has happened within a week, or more than a year ago). We refer to these models as "full feature enhanced baselines", similar to Razavian *et al.* [14].

We fitted the model on the training set separately for each hospital's data and report results when applying this model to the test set of each hospital's data. Confidence intervals were obtained using the same procedure outlined in the manuscript.

Mortality Baseline Model - aEWS

Most existing models use a small set of lab measurements, vital signs and mental status. Following this approach, for the EHR datasets, we created a model that used the most recent systolic blood pressure, heart-rate, respiratory rate and temperature in Fahrenheit (any temperature that was listed

below 90 degree Fahrenheit was converted from Celsius to Fahrenheit). Because urine output and mental status was not coded consistently between sites, we instead used the most recent white blood cell count, hemoglobin[15], sodium[16], creatinine[17,18], troponin[19–21] lactate oxygen saturation, oxygen source, glucose, calcium, potassium, chloride, blood urea nitrogen (BUN), carbon dioxide, hematocrit, platelet, magnesium, phosphorus, albumin, aspartate transaminase (AST), Alkaline Phosphatase, Total Bilirubin, International Normalized Ratio, and Absolute Neutrophil Count (ANC). All values were log transformed and standardized to have a mean of zero and standard deviation of 1 based on values for each hospital's data on the development set. We also added the hospital service and age.

Readmission Baseline Model - modified HOSPITAL score

We created a modified HOSPITAL score[22] that included the most recent value of sodium and hemoglobin log transformed and standardized (to mean 0 and standard deviation of 1) based on values for each hospital's data on the development set, binary indicators for hospital service, a binary indicator for the occurrence of any CPT codes during the hospitalization, a binary indicator for the hospitalization lasting at least 5 days, prior hospital admissions in the past year discretized to 0,1, 2-5 and >5, and admission source.

Length of Stay Baseline Model - modified Liu

We created a baseline model similar to those created using electronic health record data for general hospital populations[23]. We created a lasso logistic model with the following variables: age, gender, prior HCC codes in the timeline (counts for each one), the principal diagnosis coded as a CCS, hospital service, and the most recent lab value of each possible lab used in the mortality baseline model.

Results of full feature baselines

For the full feature simple baselines, for predicting inpatient mortality at 24 hours after admission, the AUROC was 0.93 (95%CI 0.91-0.94) for Hospital A and 0.90 (95%CI 0.88-0.92) for Hospital B. For predicting unexpected readmissions within 30-days the AUROCs at discharge were 0.74 (95%CI 0.73-0.76) for Hospital A and 0.73 (95%CI 0.72-0.74) for Hospital B. For long length-of-stay at 24 hours after admission, the AUROC was 0.83 (95%CI 0.82-0.84) for Hospital A and 0.81 (95%CI 0.80-0.82) for Hospital B.

For the full feature enhanced baselines, for predicting inpatient mortality at 24 hours after admission, the AUROC was 0.93 (95%CI 0.92-0.95) for Hospital A and 0.91 (95%CI 0.89-0.92) for Hospital B. For predicting unexpected readmissions within 30-days the AUROCs at discharge were 0.75 (95%CI 0.73-0.76) for Hospital A and 0.75 (95%CI 0.74-0.76) for Hospital B. For long length-of-stay at 24 hours after admission, the AUROC was 0.85 (95%CI 0.84-0.85) for Hospital A and 0.83 (95%CI 0.83-0.84) for Hospital B.

The results of the full-feature simple baseline are in-between the limited-feature baseline and the full-feature enhanced baseline. The results of the full-feature enhanced baseline are generally in-between the full-feature simple baseline and our deep-learning models.

Supplemental Table 1: Prediction accuracy of each task of deep learning model compared to baselines

	Hospital A	Hospital B
**Inpatient Mortality, AUROC[1](95% CI)**		
Deep learning 24 hours after admission	**0.95**(0.94-0.96)	**0.93**(0.92-0.94)
Full feature enhanced baseline at 24 hours after admission	0.93 (0.92-0.95)	0.91 (0.89-0.92)
Full feature simple baseline at 24 hours after admission	0.93 (0.91-0.94)	0.90 (0.88-0.92)
Baseline (aEWS[2]) at 24 hours after admission	0.85 (0.81-0.89)	0.86 (0.83-0.88)
**30-day Readmission, AUROC (95% CI)**		
Deep learning at discharge	**0.77**(0.75-0.78)	**0.76**(0.75-0.77)
Full feature enhanced baseline at discharge	0.75 (0.73-0.76)	0.75 (0.74-0.76)
Full feature simple baseline at discharge	0.74 (0.73-0.76)	0.73 (0.72-0.74)
Baseline (mHOSPITAL[3]) at discharge	0.70 (0.68-0.72)	0.68 (0.67-0.69)
**Length of Stay at least 7 days AUROC (95% CI)**		
Deep learning 24 hours after admission	**0.86**(0.86-0.87)	**0.85**(0.85-0.86)
Full feature enhanced baseline at 24 hours after admission	0.85 (0.84-0.85)	0.83 (0.83-0.84)
Full feature simple baseline at 24 hours after admission	0.83 (0.82-0.84)	0.81 (0.80-0.82)
Baseline (mLiu[4]) at 24 hours after admission	0.76 (0.75-0.77)	0.74 (0.73-0.75)

[1] Area under the receiver operator curve
[2] Augmented early warning score
[3] Modified HOSPITAL score
[4] Modified Liu score

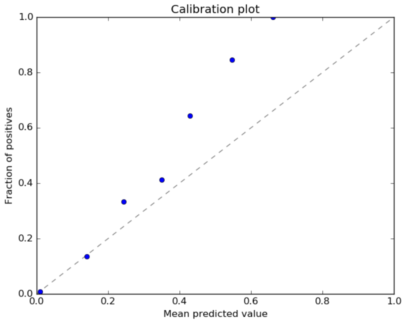

(a) Calibration curve for inpatient mortality predicted at 24 hours into hospitalization for hospital A

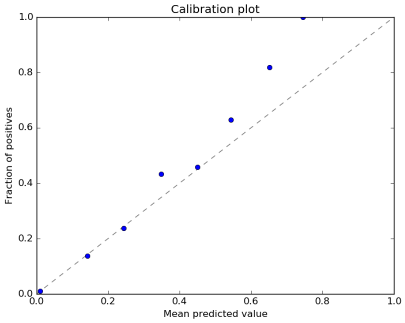

(b) Calibration curve for inpatient mortality predicted at 24 hours into hospitalization for hospital B

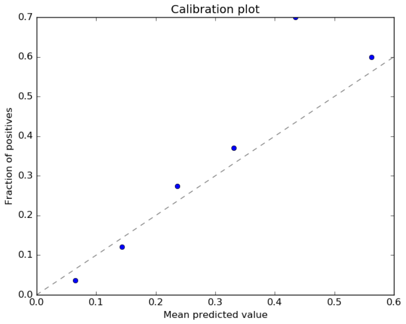

(c) Calibration curve for readmission predicted at discharge for hospital A

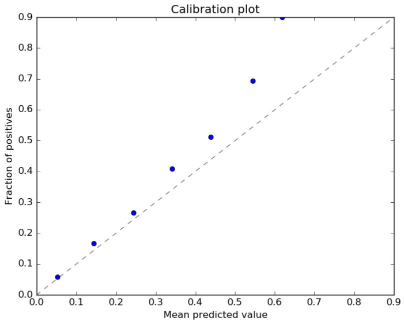

(d) Calibration curve for readmission predicted at discharge for hospital B

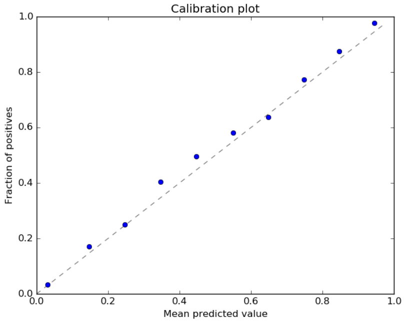

(e) Calibration curve for long length of stay predicted at 24 hours into hospitalization for hospital A

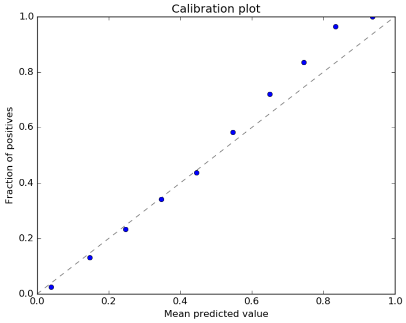

(f) Calibration curve for long length of stay predicted at 24 hours into hospitalization for hospital B

Supplemental Figure 2: Calibration curves for deep learning models

References

1. 2016 Measure updates and specifications report: hospital-wide all-cause unplanned readmission — version 5.0. *Yale–New Haven Health Services Corporation/Center for Outcomes Research & Evaluation* (May 2016).
2. *CMS' ICD-9-CM to and from ICD-10-CM and ICD-10-PCS Crosswalk or General Equivalence Mappings* `http://www.nber.org/data/icd9-icd-10-cm-and-pcs-crosswalk-general-equivalence-mapping.html`. Accessed: 2017-7-21.
3. Hochreiter, S. & Schmidhuber, J. Long Short-Term Memory. *Neural Comput.* **9,** 1735–1780 (Nov. 1997).
4. Krueger, D. *et al. Zoneout: Regularizing rnns by randomly preserving hidden activations* in *International Conference on Learning Representations* (2017).
5. Gal, Y. & Ghahramani, Z. *A theoretically grounded application of dropout in recurrent neural networks* in *Advances in neural information processing systems* (2016), 1019–1027.
6. Duchi, J., Hazan, E. & Singer, Y. Adaptive Subgradient Methods for Online Learning and Stochastic Optimization. *J. Mach. Learn. Res.* **12,** 2121–2159. ISSN: 1532-4435 (July 2011).
7. Kingma, D. P. & Ba, J. *Adam: A method for stochastic optimization* in *International Conference on Learning Representations* (2015).
8. Bahdanau, D., Cho, K. & Bengio, Y. Neural Machine Translation by Jointly Learning to Align and Translate. arXiv: `1409.0473` (2014).
9. Goodfellow, I., Bengio, Y. & Courville, A. *Deep Learning* (MIT Press, 2016).
10. Golovin, D. *et al. Google Vizier: A Service for Black-Box Optimization* in *Proceedings of the 23rd ACM SIGKDD International Conference on Knowledge Discovery and Data Mining* (ACM, 2017).
11. Zhou, H., Della, P. R., Roberts, P., Goh, L. & Dhaliwal, S. S. Utility of models to predict 28-day or 30-day unplanned hospital readmissions: an updated systematic review. *BMJ Open* **6,** e011060 (2016).
12. Smith, M. E. B. *et al.* Early Warning System Scores for Clinical Deterioration in Hospitalized Patients: A Systematic Review. *Ann. Am. Thorac. Soc.* **11,** 1454–1465 (2014).
13. Lu, M., Sajobi, T., Lucyk, K., Lorenzetti, D. & Quan, H. Systematic review of risk adjustment models of hospital length of stay (LOS). *Med. Care* **53,** 355–365 (Apr. 2015).
14. Razavian, N. *et al.* Population-level prediction of type 2 diabetes from claims data and analysis of risk factors. *Big Data* **3,** 277–287 (2015).
15. Kalra, P. R. *et al.* Hemoglobin and Change in Hemoglobin Status Predict Mortality, Cardiovascular Events, and Bleeding in Stable Coronary Artery Disease. *Am. J. Med.* (June 2017).
16. Biggins, S. W. *et al.* Serum sodium predicts mortality in patients listed for liver transplantation. *Hepatology* **41,** 32–39 (Jan. 2005).
17. Bucaloiu, I. D., Kirchner, H. L., Norfolk, E. R., Hartle 2nd, J. E. & Perkins, R. M. Increased risk of death and de novo chronic kidney disease following reversible acute kidney injury. *Kidney Int.* **81,** 477–485 (Mar. 2012).

18. Lafrance, J.-P. & Miller, D. R. Acute kidney injury associates with increased long-term mortality. *J. Am. Soc. Nephrol.* **21,** 345–352 (Feb. 2010).

19. Waxman, D. A., Hecht, S., Schappert, J. & Husk, G. A model for troponin I as a quantitative predictor of in-hospital mortality. *J. Am. Coll. Cardiol.* **48,** 1755–1762 (2006).

20. Kim, L. J. *et al.* Cardiac troponin I predicts short-term mortality in vascular surgery patients. *Circulation* **106,** 2366–2371 (Oct. 2002).

21. James, P. *et al.* Relation between troponin T concentration and mortality in patients presenting with an acute stroke: observational study. *BMJ* **320,** 1502–1504 (June 2000).

22. Donzé, J., Aujesky, D., Williams, D. & Schnipper, J. L. Potentially avoidable 30-day hospital readmissions in medical patients: derivation and validation of a prediction model. *JAMA Intern. Med.* **173,** 632–638 (Apr. 2013).

23. Liu, V., Kipnis, P., Gould, M. K. & Escobar, G. J. Length of stay predictions: improvements through the use of automated laboratory and comorbidity variables. *Med. Care* **48,** 739–744 (Aug. 2010).
